# Editorial: Innovations in teaching and learning for Health Professions Educators

**DOI:** 10.3389/fmed.2025.1611578

**Published:** 2025-05-20

**Authors:** Roger A. Edwards, Bobbie Ann Adair White, Ardi Findyartini

**Affiliations:** ^1^Department of Health Professions Education, MGH Institute of Health Professions, Boston, MA, United States; ^2^Department of Medical Education, Faculty of Medicine, Universitas Indonesia, Central Jakarta, Indonesia

**Keywords:** educational innovation, faculty professional development, educational research, educational training, educational research methods, simulation, artificial intelligence, health professions education

## Introduction

The health professions continue to evolve and change rapidly as more opportunities and challenges emerge (1). Hence, health professions educators are required to be adaptive and nimble in their creation and adoption of teaching and learning innovations (2–4). Scholarship related to best practices and identification of innovations is difficult because the same innovations have proven to be both engaging and burdensome (2). Simulation-based education continues to be innovative with new delivery modalities including distance simulation, while continuing to focus on effectiveness in how health professions educators are taught (5–7). However, gaps around effectiveness of training and development for health professions persist (2). Faculty effectiveness, especially as it relates to educational innovation adoption, is difficult to measure; and demonstration of related competencies is in its infancy.

This Research Topic encompasses state-of-the-art examples of scholarship in health professions education related to the awareness and appropriate adoption of innovation, which is broadly defined as an idea, practice, technology, and know-how (8). Evidence about the current state of emerging innovations including artificial intelligence, effectiveness of reflections or systems thinking as core aspects of learning, and evidence about the competencies needed for teaching in our evolving environments are included in this Research Topic. The 17 articles include quantitative and qualitative data collection, secondary data analyses, literature reviews, curriculum and assessment tool development. Covering a spectrum of health professions including medicine, nursing, pharmacy, physical therapy, and others, they reflect work conducted around the world, including US, Asia (China, Singapore), Australia, Middle East (Qatar, Saudi Arabia), and Europe (Germany). Additionally, this Research Topic includes participant samples across career stages from early trainees to continuing professional development for mid-career professionals. This Innovations in Health Professions Education Editorial organizes and highlights the articles into the following themes: (1) artificial intelligence/machine learning/computer-based training; (2) competencies and assessment tools; (3) reflections as a core aspect of learning; and (4) systems thinking as a core aspect of learning.

## Artificial intelligence/machine learning/computer-based training

From undergraduate medical education to continuing professional development, the use of AI and other technological innovations have revolutionized the ways healthcare professionals learn and practice. For best implementation, it is pertinent that the use of any forms of technology in healthcare education considers the readiness of the users—as reported by Gharib et al.. Students tend to be more eager to adopt new technologies whereas educators on the contrary express more reluctance due to workload and technology efficacy concerns.

Large language models (LLM) such as GPT-4 represent perhaps one of the more transformative technological advancement in health professions education. Nevertheless, we must critically examine and mitigate the risks of using AI such as algorithmic bias, overreliance, plagiarism, misinformation, inequity, privacy, and copyright concerns in health professions education through integral and systematic use (9). These recommendations align with the structured literature review on the use of LLM in healthcare simulation provided in the series by Maaz et al.. The paper offers guidance on prompt design for healthcare simulations which is particularly useful for clinical scenario development, OSCE station creation, simulated person scripting, and debriefing facilitation.

Despite the potentials of AI use in postgraduate medical education in helping residents develop critical thinking skills with algorithmic reasoning (10), careful consideration of their limitations, and the imperative to maintain human oversight in clinical education should always be incorporated. Yang et al. proposes a new paradigm of data-intensive clinical research as a pivotal strategy in medical advancement. This approach encompasses multidisciplinary integration needs, high-quality faculty, learning method transformations, assessment system updates, and awareness toward ethical concerns.

Finally, one of the published papers in this Research Topic by Janumpally et al. offers five major potential impacts of generative AI in graduate medical education which include reduced EHR documentation burden, enhanced clinical simulations, personalized educational experiences, supported research and data analysis, and improved clinical decision making. As Masters et al. (11) emphasize, technology should augment rather than replace the human elements of healthcare education—mentorship, role modeling, and professional identity formation remain irreplaceable components of developing compassionate, competent healthcare professionals. Moving forward, a balanced approach that leverages technological advantages while preserving essential human interactions will be crucial for preparing healthcare professionals to practice effectively in increasingly complex and technologically-mediated healthcare environments.

## Competencies and assessment tools

In medical and health professions education, assessment is a hot topic on both macro and micro levels. On a macro level, programs strive to meet assessment requirements and standards set by accreditation bodies. Competency-based learning objectives and assessments are imperative as programs move in that direction. The dilemma is how to assess demonstrated competency and behaviors. In the article by Williams et al., there are seven literature supported tips for integrating behavioral assessments, guiding transition and compliance around competency-based medical education. The macro literature also includes a systematic review of clinical learning environment tools within nursing education (Xu et al.), highlighting nine instruments that measure the clinical learning environment and can be used for future research.

On a micro or programmatic level, programs have created assessment tools. Mohamed et al. created an assessment tool through action research to monitor weekly clinical skills progress in their nursing students and to ensure constructive feedback from key stakeholders like the nursing faculty and students. Although not directly about competency and assessment, the final article in this section by Zhang et al. highlighted methods [virtual reality technology combined with “Bridge-in, Objectives and Outcomes of Learning, Pre-assessment, Participatory learning, Post-assessment, and Summary” (BOPPPS)] that improved learning which could impact competency. In their study, they conducted a traditional educational intervention with experimental and control groups finding that the experimental group, receiving education with virtual reality technology and BOPPPS demonstrated better outcomes. These articles highlighted the innovative ways health professions educators are attempting to improve education and assessment internationally.

## Reflections as core aspect of learning

Varied applications of reflection have been used as learning and assessment tools in the health professions impacting things like empathy and comfort with nuanced topics (12, 13). As a teaching and learning tool, reflection enables learning to move from theory to application (14). Several submissions in this Research Topic included reflection with some using reflection in teaching, others in assessment, and some in their methodology of investigation. A cross-sectional study by Kinney et al. found reflection on digital recordings of patient encounters for physical therapy students served as a useful educational modality. Additionally, they recommended the need for agreed upon best practices around student reflection such as frameworks or guidelines for reflection. Schmude et al. utilized an e-portfolio to assess their personal and professional curriculum within their medical doctorate. Part of their e-portfolio included reflection on learning which necessitated faculty training on previously published tools (15, 16).

In Berri et al., reflection was used as part of a post-test within an educational intervention about chemical denervation for medical residents. Their use of reflection included student self-assessment on knowledge and student intentions for future implementation. Another study used reflective exercises in a longitudinal multi-profession educational intervention on empathy. First-year dentistry, medicine, nursing and pharmacy students participated in reflective exercises, requiring students to be personally aware of their experiences (Müller et al.). The last two studies in this section were less about using reflection for teaching and learning, but instead about using reflective experiences and writings to understand things like leadership and educational development. Leveraging constructivist learning theory and reflection, Dewsnap and Konatham explored a new and less resource intensive way of teaching and learning about leadership, moving away from lecture-based education. In the final study by Schumann et al. researchers used qualitative methods to identify and understand what motivates health professionals to participate in additional training around education, finding that intrinsic motivation, competence, collaboration and mentorship were some of the components that motivated them to pursue additional education.

## Systems thinking as a core aspect of learning

Health professions education occurs at the intersection of two very complex systems (health care and education) with diverse, interacting components reflecting messy, non-linear relationships and substantial uncertainty (2). Systems thinking and complex adaptive systems (CAS) are useful frameworks for considering how stakeholders can be considered in planning and implementing health professions education programs (20) and professional development programs to train faculty (17). In this Research Topic, Cola et al. explicitly incorporated a systems thinking approach to effectively teach U.S. FDA regulatory processes to students, faculty, and staff at multiple schools of medicine and engineering in northeastern Ohio. They “highlight the effectiveness of an interdisciplinary and transdisciplinary approach to teaching and learning for biomedical education, specifically in preparing participants for the complexities of FDA regulatory processes and biomedical entrepreneurship”. They employed the principles of andragogy (18, 19) to create an applied learning experience that is relevant to a diverse mix of students. Wells et al. also described a transdisciplinary dual degree program based on andragogical principles that chronologically, experientially, and conceptually integrates biomedical topics with the goal of achieving “transformative educational outcomes”. They noted the importance of faculty willing to go beyond their disciplines' comfortable boundaries and teach educational materials “transdisciplinarily” in order to create “physicianeers”. As noted above, Yang et al. also touch on these themes in creating graduate educational experiences for data intensive machine learning and artificial intelligence applications.

## Conclusion

The field of health professions education faces enormous challenges as we strive to keep up with emerging technology capabilities and disparate learning preferences across the spectrum of career stages. Competencies related to the use of AI-assisted clinical decision support are only beginning to be considered and studied (20, 21), posing challenges for educators who themselves need training about how to effectively utilize innovations in the service of better learning experiences and achievement of required learning outcomes. Consistently adopting systems thinking approaches combined with reflections are valuable ways to evaluate health professions education innovations. Methodological challenges associated with studying educational innovations and measuring attainment of competencies (e.g., related to AI) remain critical research needs. We hope this collection of articles inspires readers to embark on new scholarship as we learn together.
